# Routine Pre-operative Laboratory Evaluation: Our Experience at an Obstetric and Gynaecological Pre-anaesthetic Check-Up Clinic

**DOI:** 10.7759/cureus.64112

**Published:** 2024-07-08

**Authors:** Sifna Tahir, Altaf A Mir, Imran A Siddique, Abdul Hameed

**Affiliations:** 1 Anaesthesia, Critical Care and Palliative Medicine, Government Medical College, Srinagar, IND; 2 Biochemistry, All India Institute of Medical Sciences, Raebareli, Raebareli, IND; 3 Biochemistry, Employee State Insurance Corporation Medical College, Hyderabad, IND

**Keywords:** pre-operative laboratory investigations, american society of anaesthesiologists (asa), elective surgery, peri-anaesthetic management, pre-anaesthetic check-ups

## Abstract

Introduction

Laboratory testing is done before surgery to identify body abnormalities that cannot be detected through clinical evaluation alone. Patients going in for low- or intermediate-risk surgeries are often encouraged to undergo a battery of tests as usual. This cross-sectional observational study evaluated the status of routine pre-operative laboratory tests in American Society of Anaesthesiologists (ASA) Grade I, II, and III adults undergoing elective surgery at a maternity hospital, as well as the impact of these tests on the outcome of the pre-anaesthetic check-up (PAC).

Methods

The present observational study was conducted on 500 patients scheduled for elective surgery under anaesthesia. The procedures included routine gynaecological and obstetric surgeries like abdominal hysterectomy, suction evacuation, laparotomy for ectopic pregnancy, diagnostic biopsy, and lower segment caesarean section, among others. A designated anaesthesiologist gathered information from the completed PAC sheets. As per the standard departmental policy, each patient underwent a clinical examination and routine investigations at the PAC clinic. In addition to demographic and other variables, laboratory test results and any peri-operative interventions performed due to abnormalities were assessed. Investigations already done, asked by anaesthesiologists, and referral services sought were noted. The impact of these investigations on anaesthetic decision-making was noted. Data were expressed in frequencies and percentages and statistically analysed using INSTAT software (GraphPad Prism Software Inc., La Zolla, USA).

Results

The age and weight of the patients range from 20 to 70 years and 55 to 95 kg. Most patients belonged to ASA Grade II (n=348, 69.6%). Hypothyroidism was the most common abnormal finding (n=122, 22.4%). Anaemia, hypertension, and diabetes were detected in n=8 (1.6%), n=82 (16.4%), and n=34 (6.8%) of patients, respectively. In 488 (97.6%) patients, one or more of the investigations from the list were pending. Based on the results of various preoperative laboratory investigations, 87 (17.4%) patients were advised of multiple specialty opinions before surgery. A total of 453 (90.6%) patients attending the clinic were recommended to review their PACs after their pending investigations and specialist consultations were completed. At the same time, n=41 (8.2%) was found to be fit for surgery, and n=6 (1.2%) was found unfit for surgery and was postponed.

Conclusions

The incidence of tests with abnormal results was a little high in our study. One reason could be that a particular group of patients is included in the study. Preoperative laboratory investigations substantially increase the costs. Not many patients with abnormal tests may require changes in their peri-anaesthetic management. Nonetheless, laboratory tests can help ensure the patient is in an ideal preoperative condition. Pre-operative laboratory investigations should be advised on a case-by-case basis to avoid inconveniencing the patient, delaying the surgical procedure, and driving up the cost of surgical treatment.

## Introduction

Prior to surgery, every patient undergoes a pre-operative evaluation. Laboratory testing is used to detect problems in the body that couldn't be found by a physical and clinical examination alone. Additionally, it may be done to determine the degree to which the underlying clinical condition has caused a derangement in the patient's physiology. Some investigations are carried out during surgical workups. Also, as a part of a pre-anaesthetic check-up (PAC), a battery of tests is often advocated as a routine, even in healthy patients scheduled for low- or intermediate-risk surgery. Patients are assigned one of six grades based on their physical status, as determined by the American Society of Anaesthesiologists (ASA) [1]. It has been realised, though, that many of the investigations are of minimal benefit. Numerous researchers have thus questioned the necessity of conducting these investigations [2-3]. The existing research on pre-operative testing and its cost implications has primarily originated in developed countries [4-5], with few studies conducted in our population [6-7]. In light of the fact that the healthcare industry is attempting to reduce additional expenditures caused by these investigations in an effort to contain rising costs, it is prudent to exercise caution when requesting these laboratory tests during PAC. The availability of numerous international guidelines can aid decision-making [8-9]. The guidelines established by the National Institute for Clinical Excellence (NICE) are one such resource [9]. As stated in the consensus of these guidelines, unnecessary tests may occasionally result in patient injury, additional financial burden, or surgical postponement [10-11]. However, due to limited access to healthcare in India, it is possible that these guidelines may not entirely apply to our population. The present study aimed to evaluate the status of routine preoperative laboratory investigations ordered in a referral maternity hospital of a medical college hospital in North India for ASA Grade I, II, and III adults undergoing elective surgery. The primary objective was to evaluate the prevalence of abnormal test results by looking at the frequency of both new and comorbid findings. The secondary objective included the study of the impact of the investigations on perioperative management.

## Materials and methods

The present cross-sectional observational study was conducted at a tertiary care maternity hospital. Patients of the female sex, aged 20 to 70 years, belonging to any ASA grade and who attended a PAC clinic and were scheduled for elective gynaecologic and obstetric surgery under general, regional, or combined general-regional anaesthesia were recruited. Patients under 20 years of age, more than 70 years of age, or undergoing emergency surgery were excluded from the study. Examples of surgeries selected for the study were lower segment caesarean section (LSCS), abdominal hysterectomy, colporrhaphy, dilation and curettage, cervical biopsy, endometrial biopsy, laparotomy, myomectomy, cyst excision, polypectomy, cervical cerclage, hysteroscopy, vaginal hysterectomy, hysteroscopic removal, resuturing, laparoscopy, and tubal ligation. Each patient attended the PAC clinic, where a fixed consultant anaesthesiologist did a thorough clinical evaluation (history taking and physical examination) for risk stratification. The anaesthesiologist collected the data by screening the investigation files and the completed PAC record sheets. The surgical team already did the investigations before sending the patient to PAC OPD for evaluation and risk stratification, and the investigations were inquired about/noted by anaesthesiologists in the PAC record sheet, and referrals sought were noted. Routine blood investigations were conducted according to institutional policy. They included complete liver function tests (LFTs), renal function tests (RFTs), coagulograms, serology, chest X-rays, blood grouping, complete blood count (CBC), erythrocyte sedimentation rate (ESR), and blood sugar. Thyroid function tests (TFTs), manual platelet count, echocardiography, and glycated haemoglobin (HbA1c) were also requested if required. A specially designed proforma was filled for each patient. This included name, Medical Records Department number, diagnosis, procedure, co-morbidity, pending investigations, special requirements, consultation, body weight, ASA grade, risk status, and PAC outcome. Consultation is defined as a referral to a specialist for any peri-operative complications related to abnormal test results, clearance to give anaesthesia, or a change in anaesthetic plan. The data were expressed in absolute numbers and percentage scales.

## Results

A total of 500 female patients scheduled for elective gynaecologic and obstetric surgical procedures were evaluated. Most patients were adults and in ASA physical Gtatus II (n=348, 69.6%). The number of patients in ASA Grades I and III was n=149 (29.8%) and n=3 (0.6%), respectively. The age and weight of the patients ranged from 20-70 years and 55-95 kg. The indication for surgery (diagnosis) and the surgical procedure followed are shown in Table 1.

**Table 1:** Details of patient diagnosis and the nature of elective surgeries to be performed on the patients IUD: intrauterine death, IUCD: intrauterine contraceptive device, LSCS: lower (uterine) segment caesarean section

Diagnosis	Total number	Elective surgery	Total number
Bleeding per vaginum	20	LSCS	209
Cervical prolapse	1	Abdominal hysterectomy	40
Menorrhagia	14	Colporrhaphy	4
Amenorrhoea	232	Dilation and curettage	107
IUD	2	Cervical biopsy	9
Retained products of conception	24	Endometrial biopsy	19
Ectopic pregnancy	28	Laparotomy	18
Uterine fibroid	13	Myomectomy	5
Uterine prolapse	10	Cyst excision	1
Ovarian cyst	15	Polypectomy	7
Vaginal wall cyst	3	Cerclage	2
Invasive mole	1	Hysteroscopy	20
Molar pregnancy	12	Vaginal hysterectomy	14
Post menopausal bleeding	15	Hysteroscopic removal	10
Primary infertility	14	Resuturing	8
Secondary infertility	13	Laparoscopy	20
Examination under anesthesia	1	Tubal ligation	7
Missed abortion	17		
Pain in abdomen	12		
Misplaced IUCD	10		
Wound gapping	8		
Polymenorrhagia	10		
Cervical polyp	2		
Carcinoma cervix	1		
Hematometrocolpos	1		
Vaginal prolapse	1		
Cervical fibroid	1		
Placenta accreta	1		
Tubal recanalization	1		
Tubal block	2		
Tubal ligation	7		
Vault prolapse	2		
Endometrial polyp	1		
Stress incontinence	1		
Vulvar malignancy	1		
Vulvar ulceration	1		
Endometrial biopsy	1		
Ovarian dermoid	1		

The indications for surgery ranged from misplaced intrauterine contraceptive devices (IUCD) to carcinoma of the cervix. Out of 500 patients, the majority were amenorrhoea patients, and n=232 (46.4%) were posted for LSCS. The significant others included retained products of conception (n=24, 4.8%), ectopic pregnancy (n=28, 5.6%), missed abortion (n=17, 3.4%), and ovarian cyst (n=15, 3%). Most of the surgical procedures included LSCS (n=209, 41.8%), dilation and curettage (n=107, 21.4%), abdominal hysterectomy (n=40, 8%), and laparotomy (n=18, 3.6%). Out of 500 patients, 248 (49.6%) were free from any co-morbidity. In the rest, the co-morbidities included hypothyroidism (n=112, 22.4%), hypertension (HTN) (n=60, 12%), type 2 diabetes mellitus (T2DM) (n=25, 5%), gestational HTN (n=22, 4.4%), deranged LFTs (n=22, 4.4%), and others (Table 2).

**Table 2:** Details of co-morbidity and pending investigations in the patients attending the PAC clinic PAC: pre-anaesthetic check-up, RHD: rheumatic heart disease, GDM: gestational diabetes mellitus, DVT: deep vein thrombosis, ITP: idiopathic thrombocytopenic purpura, HTN: hypertension, T2DM: type 2 diabetes mellitus, LBBB: left bundle branch block, AF: arterial fibrillation, PIH: pregnancy-induced hypertension, LFT: liver function test, RFT: renal function test, URTI: upper respiratory tract infection, ECG: electrocardiogram, APH: antepartum haemorrhage, ASD: arterial septum closure, BT/CT: bleeding time/clotting time, TFT: thyroid function test, MPC: manual platelet count, CBC: complete blood count, ESR: erythrocyte sedimentation rate, HbA1c: glycated haemoglobin, RBS: random blood sugar, FBS: fasting blood sugar

Nature of co-morbidity	Total number	Pending investigations	Total number
Hypothyroidism	112	LFT	62
RHD	4	BT/CT	239
Rectal carcinoma with metastasis	1	No investigation pending	12
GDM	9	Echocardiography	2
Severe anaemia	8	Tripple serology	201
DVT	1	ECG	59
Laparotomy	2	Chest X-ray	27
ITP	2	TFT	83
Ascites	1	MPC	33
HTN	60	All investigations pending	10
T2DM	25	CBC	31
Psoriasis	1	Electrolytes	27
LBBB with AF	1	Blood grouping	3
PIH	22	RFT	52
Bronchial asthma	6	ESR	8
No co-morbidity	248	Coagulogram	21
Rh positive	1		
Central placenta previa	1	HbA1c	3
Ebstein anomaly operated	1	RBS	20
Increased cholesterol	1	FBS	2
Deranged LFT	22		
Deranged RFT	1		
Low lying placenta	1		
Placenta covering OS	1		
Hydramnios with depression	1		
Acreta placenta	1		
Low platelets	6		
Papillary carcinoma	1		
Chronic hepatitis	2		
Carcinoma colon operated	1		
Cheekbone tumour	1		
Cholelithiasis	1		
URTI	2		
Mitral valve regurgitation	2		
Placenta previa	1		
ECG abnormality	3		
Tubercular meningitis	1		
Seizure	1		
Goiter	1		
Nephrectomy	1		
Cough and wheeze	1		
Fibroid	1		
Recurrent chest infection	1		
APH	1		
Congenital heart disease with ASD closure	1		
Migraine	1		

Among the patients included in the study, 2.4% (n=12) had completed all investigations from the list, whereas 2% (n=10) had all investigations pending. One or more tests from the list were pending for the rest of the patients. Bleeding time/clotting time (BT/CT) (n=239, 47.8%) and triple serology (n=201, 40.2%) contributed substantially to the pending investigation list. The others included in this list were TFTs (n=83, 16.6%), LFTs (n=62, 12.4%), electrocardiograms (ECG) (n=59, 11.8%), RFTs (n=52, 10.4%), and others (Table 2). From the 500 patients studied, n=234 (46.8%) had special requirements in the form of one or more units of blood, platelet-poor plasma (PPP), fresh frozen plasma (FFP), and packed red blood cells (PRBCs) for transfusion, while in n=266 (53.2%), there were no special requirements (Table 3).

**Table 3:** Details of special requirements and referral consultations for PAC clinic patients PAC: pre-anaesthetic check-up, PPP: platelet-poor plasma, FFP: fresh frozen plasma, PRBCs: packed red blood cells

Special requirements	Total number	Referral consultations	Total number
1 unit of blood	184	Physician consultation	47
2 units of blood	30	Cardiology consultation	16
3 units of blood	2	Hematology consultation	8
4 units of blood	1	Endocrinology consultation	8
5 units of blood	1	Dermatology consultation	1
PPPs units	1	Gastroenterology consultation	1
FFPs units	4	Psychiatric consultation	1
2 units of PRBCs	1	Pulmonary consultation	2
1 unit of PRBCs	10	Neurosurgery consultation	1
No special requirement	266	ENT consultation	2
		No need for any clearance/consultation	413

Based on the results of various preoperative laboratory investigations, n=87 (17.4%) patients were advised of multiple specialty opinions before surgery. The referral consultations included physician consultations (n=47, 9.4%), cardiology consultations (n=16, 3.2%), and others (Table 3). In n=413 (82.6%), no such clearance was required. Of all the patients involved in the study, a total of 453 (90.6%) patients who visited the clinic were advised to review their PAC when their pending investigations and specialist consultations were done. Meanwhile, n=41 (8.2%) was declared fit for the surgery, and n=6 (1.2%) was found unfit for the surgery and was hence deferred (Table 4). The PAC review was conducted at the same clinic or just before the surgery, depending on the referral consultation.

**Table 4:** Details of the ASA grade and PAC outcome of the patients ASA: American Society of Anesthesiologists, PAC: pre-anaesthetic check-up

ASA grade	Total number	PAC outcome	Total number
ASA I	149	Review	453
ASA II	348	OK	41
ASA III	3	Case postponed	6

## Discussion

Almost all patients presented with routine pre-operative tests done as advised by surgeons. The objective of PAC is to collect data on the patient and develop an anaesthetic strategy to provide anaesthesia smoothly with little or no complications during the perioperative period [12]. Performing routine pre-operative investigations can potentially help identify previously undetected conditions requiring treatment before surgery or a change in anaesthesia management. However, it is essential to note that there is a risk of obtaining false positive results, which may result in unnecessary, expensive, and potentially harmful treatments or additional investigations that can delay the surgery [13]. According to the ASA, there is no need for regular laboratory or diagnostic screening tests during the pre-anaesthetic evaluation of patients [14]. The patient's medical history should determine the pre-operative investigations, thorough physical examination, assessment of the risks associated with the surgery, and use of clinical expertise to make informed decisions [15]. Anesthesiologists exhibit a higher level of rationality when determining the necessary criteria for conducting routine testing. Testing ordered by an anesthesiologist is more targeted and economical compared to testing performed by a surgeon [16]. According to a cost analysis study, anesthesiologists' selective ordering of investigations decreased the number of tests and their associated costs by almost 25% [16]. If a consultant evaluated and ordered the tests, the cost would decrease by 41%. Earlier research has found that a cost reduction occurs due to patient-directed investigations [4,17-18].

Pre-anaesthetic investigative practices vary considerably. They differ according to the surgical procedure, equipment, and setup at hand to manage any intraoperative or postoperative complications. Likewise, they depend on the population's socioeconomic position, the timing of the presentation, and the nature of the disease being treated. The current study identified distinct aberrant test results, including severe anaemia (n=8, 1.6%), hypothyroidism (n=112, 24.4%), gestational diabetes mellitus (GDM) (n=9, 1.8%), T2DM (n=25, 5.1%), low platelet count (n=6, 1.2%), deranged LFT (n=22, 4.4%), and deranged KFT (n=1, 0.2%). This suggests that, as prior research has demonstrated, failing to account for those could result in cancellation or postponement [19-20]. The prevalence of hypothyroidism, diabetes, and severe anaemia among the patients in this study suggests that conducting tests based on patient history and co-morbidity is unlikely to overlook these abnormalities, even if we were to deviate from routine practice. The incidence of tests with abnormal results was a little high in our study. One reason could be that a particular group of patients is included in the study. Additionally, the current research was carried out in a public hospital established in the Himalayan region of a developing nation, with the majority of the patients belonging to a lower socioeconomic status. The primary abnormalities observed were diabetes, anaemia, and thyroid hormone abnormalities. In the majority of patients, the mere occurrence of an aberrant test result during routine testing does not significantly alter the approach to anaesthesia or perioperative treatment. However, laboratory tests can help ensure the patient is in an ideal preoperative condition. The current study's findings suggest that patients with co-morbidity exhibit a higher prevalence of aberrant test results, which in turn leads to an increased number of significant impacts. Therefore, it is imperative to conduct further investigations tailored to their co-morbidity. Compared to those without, these abnormalities have a more substantial impact in terms of consultation, referrals, or additional testing on patients with co-morbidities. In the current study, it was, therefore, necessary to refer patients to other physicians and specialists to modify the treatment of comorbid diseases and the management of anaesthesia. We observed that many patients were referred to cardiologists, physicians, haematologists, and endocrinologists for evaluation of patients scheduled for surgical procedures based on the age and nature of the procedure. This instrumental investigation was necessary based on the patient's clinical condition and the seriousness of the pre-existing co-morbidities.

The ASA statement suggests that pre-operative tests may be necessary to detect or diagnose a disease or disorder that could impact the administration of anaesthesia during the perioperative period. It also verifies or examines a condition, ailment, medical treatment, or alternative therapy that may impact perioperative anaesthesia care [8]. The statement also indicated that specific clinical indicators or risk factors should be identified to determine when testing should be ordered. A pre-existing disease or medical co-morbidity is included as one of the indications [8]. Our study involved conducting routine and specialised investigations on regular patients and patients with comorbidities who were scheduled for elective surgery. We then implemented perioperative management, which included additional specific investigations, referrals to specialists, and preparation for any special requirements. After conducting a clinical examination and evaluating the available investigations during the PAC, it was determined that 453 patients (90.6%) should have their PAC reviewed after specialist referrals and investigations. Additionally, six patients (1.2%) were deemed unfit for surgery and postponed their procedures. The PAC review occurred a few days before or right before the surgery. Performing pre-operative tests, as indicated by prior research, is intended to identify concealed abnormalities and comprehensively understand current co-morbidities [8]. Nonetheless, we must consider whether or not these concealed abnormal test results substantially alter the perioperative or anaesthetic management and outcome. According to a multicentre study, pre-operative tests revealed abnormalities in 27% of the patients, with 54.5% of these aberrant test results being newly identified [21]. In the National Surgical Quality Improvement Program database, 61.6% of elective, low-risk ambulatory surgery patients had at least one abnormal test result. Despite this, surgeries were conducted on the same day [22]. A study of patients over 70 indicated abnormal test results did not predict unfavourable postoperative outcomes [23].

Both anaesthesiologists and surgeons continue to widely adhere to the old practice of doing regular pre-operative investigations prior to elective surgery [24]. This results in a large number of unnecessary or unindicated tests, as well as a significant financial burden [10-11]. More than 60% of anesthesiologists and over 70% of surgeons believe pre-operative tests will reveal a hidden problem [24]. However, we must assess if these hidden aberrant test results significantly impact perioperative or anaesthetic care and outcomes. Again, the cost-effectiveness of finding one such important case that will change how anaesthesia is managed during surgery and the result in lowering death rates must be considered. In this context, it is essential to note that patients may experience a change in their ASA physical grade from III to II after optimising an undetected disease condition [25]. As anaesthesia practices and standards continue to advance, anaesthesia-related mortality is currently relatively low. The mortality rates for ASA Grades II and III at 48 hours post-operatively are 0.002% and 0.028-0.019%, respectively [26-27].

The estimated cost savings, as determined by multiple studies, relate to implementing the NICE guidelines in the patient population. On the other hand, there might be better approaches to data interpretation and conclusion drawing in our case. When compared to developed countries, developing countries have numerous challenges when it comes to gaining access to healthcare facilities. Education and affordability are the only factors that contribute to the limited awareness of health issues, with urban populations having an advantage over rural populations. Thus, what has been deemed inappropriate in most cases may be an appropriate test in the given situation. The incidence of diabetes is increasing, and the clinical landscape is changing. Based on this, a blood glucose estimate, which is deemed superfluous in some guidelines, may be appropriate in our group of patients, who are being assessed for the first time for any pre-existing disease. Therefore, during PAC, there is a possibility that only a small number of tests will be abnormal when they are carried out in circumstances where they are not indicated. Furthermore, this would affect the anaesthetic plan, which would increase the cost, but it is worthwhile for our population. Meanwhile, conducting thorough pre-operative evaluations of patients can greatly decrease the need for needless investigations without compromising the collection of clinically valuable information and patient care. Identifying risk factors and requesting tests is essential, considering the patient's comorbid diseases and the type of surgery. For the evaluation of these patients slated for surgery, guidelines facilitate a systematic, evidence-based, and patient-centred approach. However, it is necessary to establish comparable guidelines that are founded on the characteristics of the study population.

## Conclusions

According to the findings of a comparison between local practice and the criteria established by NICE for preoperative laboratory testing, most investigations need to be more balanced. There is anticipated heterogeneity in the practice of preoperative laboratory testing between different institutes. Prior studies have predicted that conducting research at other centres or a multicentre study involving diverse geographical locations and populations will provide more elucidation. There is a lot that can be done to rationalise the practice and minimise healthcare costs while maintaining patient care quality. Implementing comprehensive pre-operative laboratory testing guidelines in clinical practice can result in substantial cost reductions by optimising the use of pre-operative tests. We need to stop routine testing and start ordering tests specific to the patient, the disease, and the patient's needs. Given the inadequate adherence of our population to routine health check-ups, it may be imperative to establish country-specific guidelines.
